# Herbal Medicine Goshajinkigan Prevents Paclitaxel-Induced Mechanical Allodynia without Impairing Antitumor Activity of Paclitaxel

**DOI:** 10.1155/2013/849754

**Published:** 2013-10-02

**Authors:** Muh. Akbar Bahar, Tsugunobu Andoh, Keisuke Ogura, Yoshihiro Hayakawa, Ikuo Saiki, Yasushi Kuraishi

**Affiliations:** ^1^Department of Applied Pharmacology, Graduate School of Medicine and Pharmaceutical Sciences, University of Toyama, Toyama 930-0194, Japan; ^2^Division of Pathogenic Biochemistry, Institute of Natural Medicine, University of Toyama, Toyama 930-0194, Japan

## Abstract

Chemotherapy-induced peripheral neuropathy is a major dose-limiting side effect of commonly used chemotherapeutic agents. However, there are no effective strategies to treat the neuropathy. We examined whether Goshajinkigan, a herbal medicine, would prevent paclitaxel-induced allodynia without affecting the anticancer action in mice. Murine breast cancer 4T1 cells were inoculated into the mammary fat pad. Paclitaxel (10 and 20 mg/kg, intraperitoneal, alternate day from day 7 postinoculation) inhibited the tumor growth, and Goshajinkigan (1 g/kg, oral, daily from day 2 postinoculation) did not affect the antitumor action of paclitaxel. Mechanical allodynia developed in the inoculated region due to tumor growth and in the hind paw due to paclitaxel-induced neuropathy. Paclitaxel-induced allodynia was markedly prevented by Goshajinkigan, although tumor-associated allodynia was not inhibited by Goshajinkigan. These results suggest that Goshajinkigan prevents paclitaxel-induced peripheral neuropathy without interfering with the anti-cancer action of paclitaxel.

## 1. Introduction

Pain in cancer patients is due to the tumor itself or due to the cancer treatment including chemotherapy [1]. The incidence of pain is 58% to 69% in patients with terminal cancer and 44% to 73% in patients receiving chemotherapeutic agents [2]. The high prevalence of debilitating pain explains the lack of effective therapies, and cancer-related pain is still a severe clinical problem. Experimentally, pain can enhance the growth and metastasis of tumor [3, 4]. Therefore, pain relief is very important for both improving the quality of life and cancer treatment.

Paclitaxel is an antimicrotubule agent, which is widely indicated to treat solid neoplasms such as ovarian, breast, and lung cancer [5, 6]. Nevertheless, the use of paclitaxel is confined by its main side effect sensory neuropathy that is characterized by cold allodynia, mechanical allodynia, spontaneous pain, shooting and burning pain, tingling, and numbness, with a stocking and glove distribution [7]. These symptoms are the most common causes for the termination or dose reduction of the treatment, potentially leading to cancer progression [8]. Moreover, the cessation of therapy occasionally does not alleviate these disabling side effects and become persistent for months or years [9]. The incidence of paclitaxel-induced peripheral neuropathy is ranging from 59% to 78% [10].

Prevention is the most recommended way to treat chemotherapy-induced neuropathy. The prerequisites of ideal prophylaxis agents are potent, have no significant side effects, and are not undermining antitumor effect of the chemotherapeutic agents [11, 12]. Several medications and vitamins have been preclinically and clinically tested for their efficacy in preventing chemotherapy-induced peripheral neuropathy, but the conflicting results have been reported [8, 13–15].

Goshajinkigan is a traditional medicine which is composed of *Rehmanniae radix*, *Achyranthis radix*, *Corni fructus*, *Dioscoreae rhizome*, *Plantaginis semen*, *Alismatis rhizome*, *Hoelen*, *Moutan cortex*, *Cinnamoni cortex*, and *Aconiti Calefactum tuber*. Goshajinkigan has ability to inhibit oxaliplatin-induced pain without weakening the antitumor activity of oxaliplatin [12, 16]. In clinical setting, Goshajinkigan has been shown to attenuate the progression of peripheral neuropathy induced by docetaxel in breast cancer patients and by paclitaxel/carboplatin in ovarian or endometrial cancer patients [17, 18]. However, there are only a few reports on its effects on paclitaxel-induced mechanical allodynia in animals [19] and no reports on the effects on malignancy-induced pain and the antitumor action of paclitaxel. Therefore, in this present study, we investigated the effects of Goshajinkigan using the mice bearing breast cancer.

## 2. Materials and Methods

### 2.1. Animals

Female BALB/c mice (Japan SLC Ltd., Shizuoka, Japan), 6 weeks of age at the start of experiments, were used. They were housed 6 per cage under controlled temperature (21–23°C) and humidity (45%–65%). The room was lighted from 7:00 am to 7:00 pm and during the behavioral test. Food and water were available *ad libitum*. The study was approved by the Committee for Animal Experiments at the University of Toyama.

### 2.2. Tumor Inoculation

 Breast cancer 4T1 cells, a mammary tumor cell line derived from BALB/c mouse, were cultured in Roswell Park Memorial Institute 1640 medium containing 10% fetal bovine serum at 37°C and in a humidified atmosphere of 5% CO_2_. The 4T1 cells (5 × 10^4^ cells/20 *μ*L) or the culture medium were inoculated into the right abdominal mammary fat pad of the mice. 

### 2.3. Drugs

Paclitaxel was purchased from Sigma (St. Louis, MO, USA) and dissolved in saline containing 10% v/v Cremophor EL (Sigma) and 10% v/v ethanol. Paclitaxel or the vehicle was injected intraperitoneally (i.p.) every other day from day 7 after tumor cell inoculation. In preliminary experiments, paclitaxel at doses of 10 and 20 mg/kg significantly inhibited tumor growth, the lower dose of 5 mg/kg did not produce a significant inhibition, and the higher dose of 40 mg/kg induced severe weight loss. Therefore, the doses of 10 and 20 mg/kg were selected. Goshajinkigan extract granules were obtained from Tsumura & Co. Ltd. (Tokyo, Japan). Goshajinkigan was dissolved in tap water and administered orally every day from day 2 after tumor cells inoculation. The dose (1 g/kg) of Goshajinkigan was selected from our preliminary experiments and the published literature on the effect of Goshajinkigan on oxaliplatin-induced sensory neuropathy [12].

### 2.4. Evaluation of Body Weight, Tumor Volume, and Tumor Weight

The body weight was measured every day using an electronic balance. The tumor size was measured every day from day 8 postinoculation by using a caliper square; the longest diameter (*a*) and the width (*b*) were measured, and tumor volume was calculated by using the formula tumor volume (mm^3^) = (*a* × *b* ^2^) ÷ 2 [20]. The weight of tumor was determined after mice being sacrificed on day 26 by taking out the tumor.

### 2.5. Behavioral Test

Mechanical allodynia was evaluated by stimulating the tumor-bearing region and the hind paw on the opposite side using a fine von Frey filament with a bending force of 0.69 mN (innocuous stimulation) [21, 22]. Responses of the tumor-bearing region to the stimulus were ranked as follows: 0, no response; 1, lifting of the hind paw; and 2, head motion toward the stimulation filament or flinching. Responses of the hind paw to the stimulus were ranked as follows: 0, no response; 1, lifting of the hind paw; and 2, flinching or licking of the hind paw. A stimulation of the same intensity was applied six times to the tumor-bearing region and the hind paw at intervals of several seconds, and the average of six values was used as the pain-related score and presented as percentage. The evaluation of mechanical allodynia was carried out before drug administration.

### 2.6. Statistical Analysis

Data are presented as mean ± standard error of the mean (SEM). Time-course data were analyzed with two-way repeated measures analysis of variance (ANOVA). Statistical significance between groups was analyzed using one-way ANOVA and post hoc Holm-Sidak multiple comparisons. *P* < 0.05 was considered significant. The statistical analyses were performed using SigmaPlot graphing and statistical software (version 11; Systat Software, Inc., Chicago, IL, USA).

## 3. Results

### 3.1. Effects of Paclitaxel and Goshajinkigan on the Volume and Weight of Tumor

**Figure 1:**
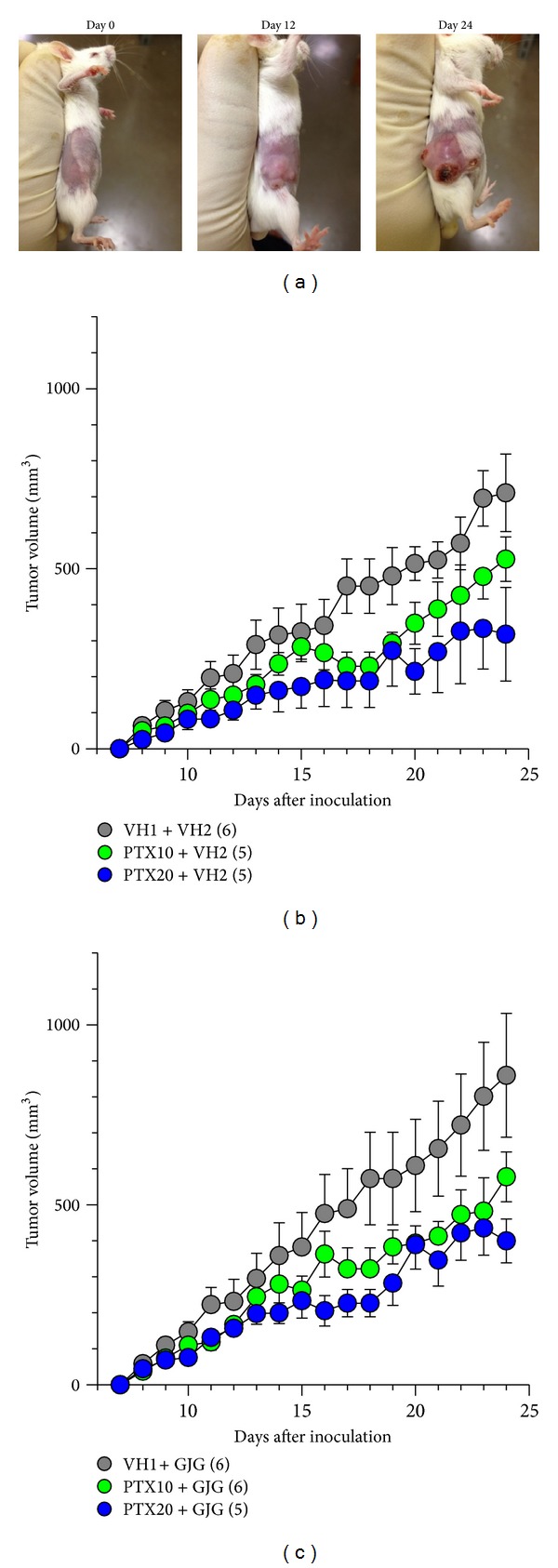
Effects of paclitaxel (PTX) and Goshajinkigan (GJG) on the growth of 4T1 cells in mice. The 4T1 cells were inoculated into the right abdominal mammary fat pad on day 0. (a) Typical example of tumor growth in the mouse breast. ((b), (c)) Time-course of the effects of PTX on the tumor growth (b) with or (c) without GJG administration. PTX (10 and 20 mg/kg) and vehicle (VH1) were injected intraperitoneally every other day from day 7 after tumor cell inoculation. GJG (1 g/kg) and vehicle (VH2) were administered orally every day from day 2 after tumor cell inoculation. Values represent the means ± SEM. Figures in parentheses indicate the number of animals. (b) Interaction between PTX treatment and time, *F*_34,221_ = 2.624, *P* < 0.001 (two-way repeated measures ANOVA). (c) Interaction between PTX treatment and time, *F*_34,238_ = 2.262, *P* < 0.001 (two-way repeated measures ANOVA).

**Figure 2:**
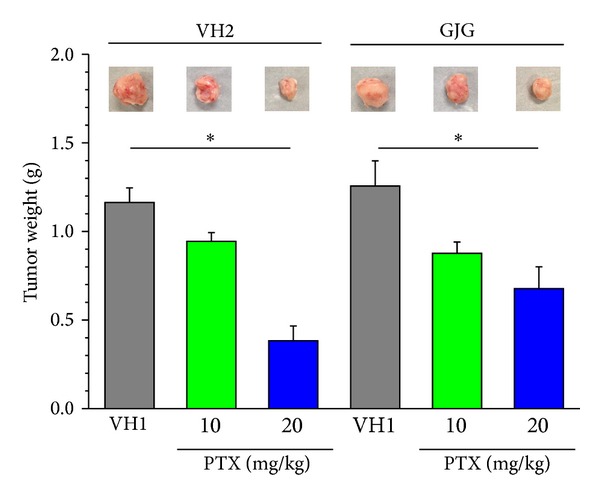
Effects of paclitaxel (PTX) and Goshajinkigan (GJG) on tumor weight in mice with breast cancer. Tumor masses were isolated from mice shown in Figure 1 on day 26 after the 4T1 cell inoculation. The photographs show typical examples of tumor mass isolated. PTX (10 and 20 mg/kg) and vehicle (VH1) were injected intraperitoneally. GJG (1 g/kg) and vehicle (VH2) were administered orally. Values represent the means ± SEM for three to six animals. *P < 0.05 (Holm-Sidak multiple comparisons).

An inoculation of 4T1 cells into the mammary fat pad of mice increased time dependently the nodule of tumor, which could be measured from day 8 postinoculation (Figure 1). Paclitaxel (10 and 20 mg/kg) inhibited the increase of tumor volume in a dose dependent manner (Figure 1(b)). When Goshajinkigan (1 g/kg) was administered daily, paclitaxel (10 and 20 mg/kg) similarly inhibited the increase of tumor volume (Figure 1(c)). On day 26 post inoculation, tumor masses were isolated from mice and weighed. Paclitaxel (10 and 20 mg/kg) reduced dose dependently tumor weight with significant inhibition at a dose of 20 mg/kg (Figure 2). In mice given repeated Goshajinkigan (1 g/kg) administration, paclitaxel also produced a dose dependent inhibition of tumor weight with significant inhibition at a dose of 20 mg/kg (Figure 2).

### 3.2. Effects of Paclitaxel and Goshajinkigan on Body Weight and Survival

The inoculation of 4T1 cells alone was not lethal to mice at least during the experimental period (Table 1). However, unexpectedly, one mouse died in each group treated with paclitaxel (10 and 20 mg/kg) alone on day 17 or 18 after the 4T1 cell inoculation (Table 1). In contrast, in the groups treated with Goshajinkigan (1 g/kg), one mouse died on day 19 postinoculation (on day 12 after the start of 20 mg/kg paclitaxel administration), and no mice died after administration of 10 mg/kg paclitaxel (Table 1).

**Figure 3:**
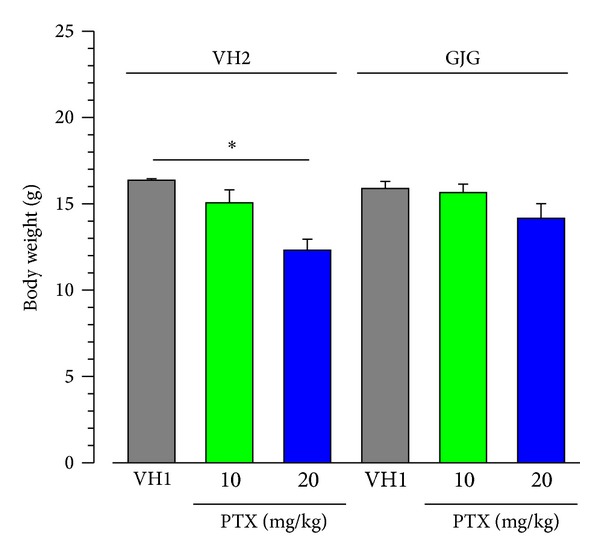
Effects of paclitaxel (PTX) and Goshajinkigan (GJG) on body weight in mice with breast cancer. The data were obtained from mice shown in Figure 1 on day 24 after the 4T1 cell inoculation. PTX (10 and 20 mg/kg) and vehicle (VH1) were injected intraperitoneally. GJG (1 g/kg) and vehicle (VH2) were administered orally. Values represent the means ± SEM for five to six animals. *P < 0.05 (Holm-Sidak multiple comparisons).

The administration of paclitaxel (10 mg/kg) alone did not decrease body weight during the observation period as compared with vehicle control, but the higher dose of 20 mg/kg significantly decreased body weight from day 14 postinoculation.  Figure 3 shows body weight on day 24 postinoculation; the administration of paclitaxel (10 and 20 mg/kg) caused a dose dependent decrease in body weight. In contrast, in the groups treated with Goshajinkigan (1 g/kg), paclitaxel (10 and 20 mg/kg) did not significantly decrease body weight during the observation period; Figure 3 shows body weight on day 24 postinoculation.

### 3.3. Effects of Paclitaxel and Goshajinkigan on Allodynia in the Hind Paw

**Figure 4:**
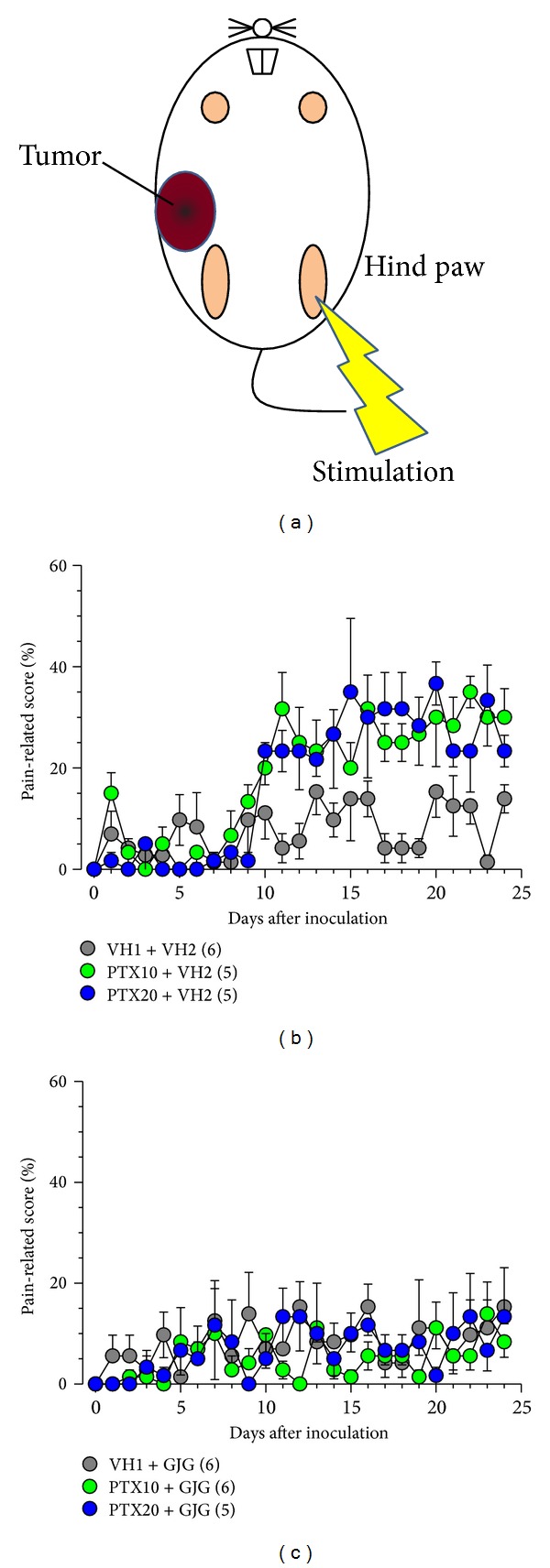
Paclitaxel- (PTX-) induced mechanical allodynia with or without Goshajinkigan (GJG) administration in mice with breast cancer. PTX (10 and 20 mg/kg) and vehicle (VH1) were injected intraperitoneally, and GJG (1 g/kg) and vehicle (VH2) were administered orally, as described in Figure 1 legend. The evaluation of pain-related responses using a von Frey filament was performed before drug administration every day. (a) The site of allodynia evaluation. ((b), (c)) Time-course of allodynia induced by PTX administration (b) with or (c) without GJG administration. Values represent the means ± SEM. Figures in parentheses indicate the number of animals. (b) Main effect of PTX treatment, *F*_2,312_ = 8.922, *P* = 0.004; interaction between PTX treatment and time, *F*_48,312_ = 2.505, *P* < 0.001 (two-way repeated measures ANOVA).

Since paclitaxel causes peripheral neuropathy, especially allodynia and dysesthesia that often occur in a “glove and stocking” distribution, we evaluated paclitaxel-induced allodynia in the hind paw in mice (Figure 4(a)). Breast cancer 4T1 cells were inoculated into the right abdominal mammary fat pad, and it is possible that pain-related responses of the ipsilateral hind paw are affected by the tumor. Therefore, we evaluated allodynia in the contralateral (left) hind paw in mice with breast cancer. Mechanical allodynia in the hind paw developed from 2 days after the start of paclitaxel (10 and 20 mg/kg) administration, although dose dependency was not obvious (Figure 4(b)). In contrast, in the groups that were given daily administration of Goshajinkigan (1 g/kg), paclitaxel (10 and 20 mg/kg) did not induce allodynia in mice with breast cancer (Figure 4(c)).

### 3.4. Effects of Paclitaxel and Goshajinkigan on Mechanical Allodynia in the Tumor Region

**Figure 5:**
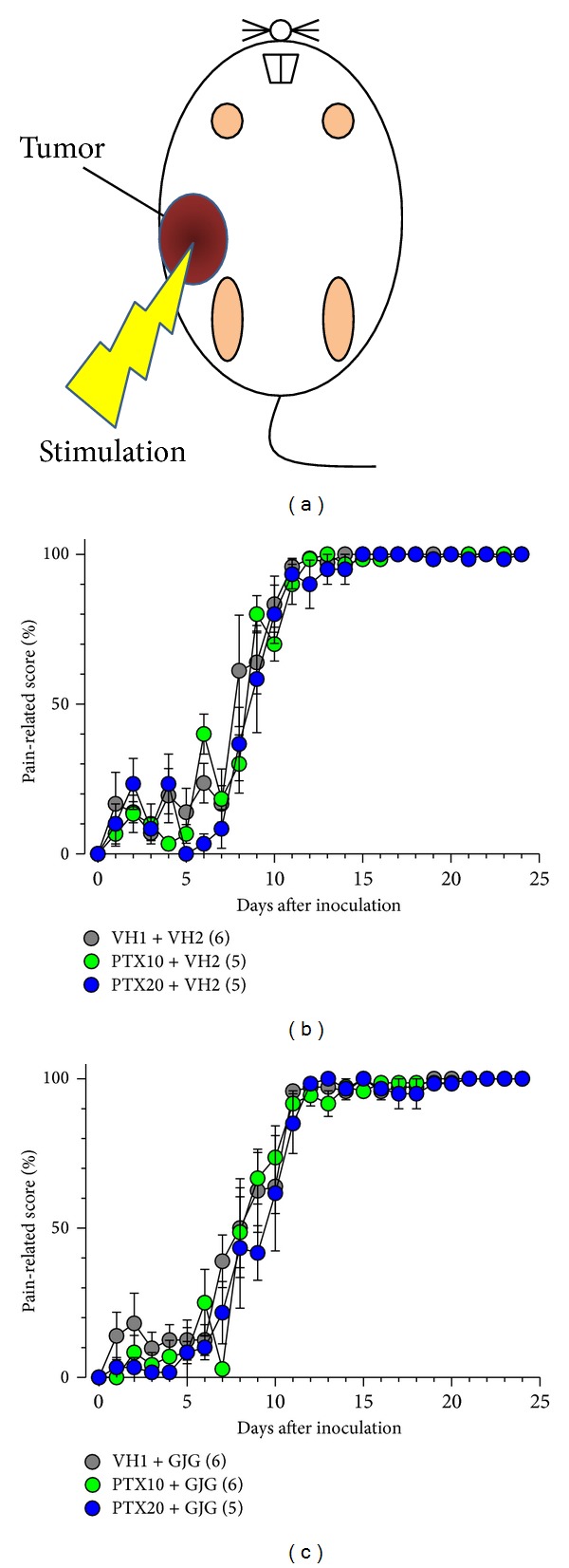
Effects of paclitaxel (PTX) and Goshajinkigan (GJG) on tumor-induced mechanical allodynia in mice with breast cancer. PTX (10 and 20 mg/kg) and vehicle (VH1) were injected intraperitoneally, and GJG (1 g/kg) and vehicle (VH2) were administered orally, as described in Figure 1 legend. The evaluation of pain-related responses using a von Frey filament was performed before drug administration every day. (a) The site of allodynia evaluation. ((b), (c)) Time-course of allodynia induced by tumor with or without PTX and GJG administration. Values represent the means ± SEM. Figures in parentheses indicate the number of animals.

Mechanical allodynia was evaluated in the region of breast cancer 4T1 cell inoculation (Figure 5(a)). Mechanical allodynia developed from day 8 postinoculation and rapidly increased to reach maximum on day 10 (Figure 5(b)). Maximal allodynia was kept at least during the observation period (day 24 postinoculation). The repeated administration of paclitaxel (10 and 20 mg/kg), Goshajinkigan (1 g/kg), or both did not affect the mechanical allodynia (Figures 5(b) and 5(c)).

## 4. Discussion

An inoculation of 4T1 cells into the mammary fat pad increased time dependently the nodule of tumor in the inoculated site in mice. Although not completely, repeated treatment with paclitaxel (10 and 20 mg/kg) significantly inhibited an increase in the tumor volume and the weight of tumor at the end of experiments. Goshajinkigan did not affect the antitumor activity of paclitaxel in mice. Goshajinkigan has also been shown not to interrupt the antitumor action of oxaliplatin on colon cancer cells [12, 16]. Thus, Goshajinkigan may not affect antitumor activity of chemotherapeutic agents.

An inoculation of 4T1 cells induced mechanical allodynia in the tumor-bearing site in mice; allodynia became apparent around day 7 postinoculation and thereafter rapidly increased for several days. Similar time-courses in tumor growth and allodynia in the tumor site were observed after melanoma cell inoculation into the hind paw in mice [21]. Although paclitaxel significantly inhibited the tumor growth, it did not affect the onset and increase of allodynia in the tumor-bearing site. Therefore, this allodynia might not be due to the increase of the tumor volume. In this context, tumor cells have been shown to release algogenic substances [23, 24]. However, it is unknown whether breast cancer cells release algogenic substances [25].

Paclitaxel induces mechanical allodynia in human [7] and in rodents [26]. Although the mechanisms are not completely understood, paclitaxel produces nerve damage by disrupting the action of microtubules necessary for axonal transport [27, 28]. Single administration of paclitaxel induces mechanical allodynia, and the effect peaks 14 days after administration and then gradually decreases [22]. In this study, repeated administration of paclitaxel elicited long-lasting allodynia in the hind paw that did not bear tumor. Repeated administration of chemotherapeutic agents may produce long-lasting allodynia [26]. Repeated administration of Goshajinkigan markedly prevented paclitaxel-induced mechanical allodynia. The mechanisms of antiallodynic activity of Goshajinkigan are still unknown. There are two conflicting reports that paclitaxel induces axonal degeneration in the sciatic nerve [29] or not [30]. However, Goshajinkigan does not prevent the oxaliplatin-induced axonal degeneration in the rat sciatic nerve, although it inhibits oxaliplatin-induced allodynia [12]. Thus, an antiallodynic activity of Goshajinkigan may not be due to the prevention of axonal degeneration, if any, after paclitaxel administration. Single paclitaxel administration gradually reduces peripheral blood flow, and the prevention of the decrease of the blood flow with limaprost alfadex, an analogue of prostaglandin E1, attenuates paclitaxel-induced mechanical allodynia [22], suggesting the involvement of the decrease of peripheral blood flow in the paclitaxel-induced mechanical allodynia. Goshajinkigan has been shown to increase blood flow and to increase nitric oxide production by activating of NO synthase [31]. Thus, it is conceivable that the improvement of peripheral blood flow is involved in antiallodynic activity of Goshajinkigan. Paclitaxel-induced mechanical allodynia is mediated by reactive oxygen species [32]. The components of Goshajinkigan have antioxidant properties [33, 34]. Thus, it is also conceivable that anti-oxidant action of Goshajinkigan is involved in the inhibition of paclitaxel-induced allodynia. 

## 5. Conclusion

Goshajinkigan prevented paclitaxel-induced allodynia without affecting the antitumor activity of paclitaxel. Thus, Goshajinkigan may be useful in the prevention of paclitaxel-induced peripheral neuropathy.

## Figures and Tables

**Table 1:** The number of survival mice.

		Days after inoculation											
		0	1	2	16	17	18	19	20	21	22	23	24
		The number of mice survived											
VH1	VH2	6	6	6	6	6	6	6	6	6	6	6	6
PTX (10)	VH2	6	6	6	6	6	5	5	5	5	5	5	5
PTX (20)	VH2	6	6	6	6	5	5	5	5	5	5	5	5
VH1	GJG	6	6	6	6	6	6	6	6	6	6	6	6
PTX (10)	GJG	6	6	6	6	6	6	6	6	6	6	6	6
PTX (20)	GJG	6	6	6	6	6	6	5	5	5	5	5	5

PTX: paclitaxel; VH1: vehicle for PTX; GJG: Goshajinkigan; VH2: vehicle for GJG.

Figures in parentheses indicate the dose (mg/kg) of PTX.

Administration schedules for PTX and GJG are shown in Figure 1.
